# Creatine supplementation enhances anti-tumor immunity by promoting adenosine triphosphate production in macrophages

**DOI:** 10.3389/fimmu.2023.1176956

**Published:** 2023-08-18

**Authors:** Zhenzi Peng, Suguru Saito

**Affiliations:** ^1^ Department of Cell Biology and Genetics, School of Basic Medical Sciences, Hengyang Medical School, University of South China, Hengyang, Hunan, China; ^2^ Division of Virology, Department of Infection and Immunity, Jichi Medical University, Shimotsuke, Japan; ^3^ Biofluid Biomarker Center, Graduate School of Medical and Dental Sciences, Niigata University, Niigata, Japan

**Keywords:** creatine, macrophages, ATP, anti-tumor immunity, CD8+ T cells

## Abstract

Creatine is an indispensable organic compound utilized in physiological environments; however, its role in immunity is still poorly understood. Here, we show that creatine supplementation enhances anti-tumor immunity through the functional upregulation of macrophages by increasing adenosine triphosphate (ATP) production. Creatine supplementation significantly suppressed B16-F10-originated tumor growth in mice compared with the control treatment. Under these conditions, intratumor macrophages polarized towards the M1 phenotype rather than the M2 phenotype, and there was an increase in tumor antigen-specific CD8+ T cells in the mice. The cytokine production and antigen-presenting activity in the macrophages were enhanced by creatine supplementation, resulting in a substantial increase in tumor antigen-specific CD8+ T cells. ATP upregulation was achieved through the cytosolic phosphocreatine (PCr) system via extracellular creatine uptake, rather than through glycolysis and mitochondrial oxidative phosphorylation in the macrophages. Blockade of the creatine transporter (CrT) failed to upregulate ATP and enhance the immunological activity of macrophages in creatine supplementation, which also impaired CD8+ T cell activity. Consequently, CrT blockade failed to suppress tumor growth in the creatine-supplemented mice. Thus, creatine is an important nutrient that promotes macrophage function by increasing ATP levels, ultimately contributing to enhanced anti-tumor immunity orchestrated by CD8+ T cells.

## Introduction

Creatine is an indispensable organic compound that is utilized in various biological activities (1). It can be synthesized from glycine and L-arginine in the kidney and liver, and is primarily stored in skeletal muscle and the brain as phosphocreatine (PCr), which conserves high-energy phosphate in the molecule (2, 3). However, the major portion of creatine in our body originates from dietary sources, such as fish and meat, and is taken up by the creatine transporter (CrT) (4). One of the most important uses of intracellular creatine is the production of adenosine triphosphate (ATP) through a specific metabolic reaction known as the PCr system (5). In this system, ATP is synthesized from adenosine diphosphate (ADP) and PCr through a reversible reaction catalyzed by creatine kinase (CK) (6). When cytosolic ATP consumption is rapid, ATP is quickly synthesized through the PCr system to provide a sufficient level of ATP for biological activities (6, 7). The concentration of creatine is tightly regulated to maintain homeostasis, although it may become dysregulated in certain physiological dysfunctions, such as high blood pressure, kidney failure, and muscle cramps (8–10). Moreover, creatine metabolism also affects disease states, as impaired creatine metabolism promotes tumorigenesis in a murine breast cancer model (11). Additionally, the loss of creatine leads to impaired brain function and severe intellectual disabilities (12). This evidence suggests that maintaining creatine homeostasis is crucial for proper biological and physiological conditions, as well as disease prevention. While the historical focus of creatine has mainly been on physiological aspects, recent studies have revealed its involvement in the regulation of both innate and adaptive immunity (13–15). These findings provide significant evidence that creatine metabolism not only regulates biochemical processes but also immune responses at both the cellular and individual levels. However, the detailed contribution of creatine to immunity is still poorly understood and characterized. In this report, we demonstrate that creatine regulates the immunological activities of macrophages by increasing intracellular ATP levels, ultimately strengthening CD8+ T cell-based anti-tumor immunity. Our findings provide a novel possibility for the use of creatine in enhancing immunity via functional upregulation of macrophages.

## Materials and methods

### Mice and tumor model

C57BL/6 mice (WT) were purchased from the Jackson Laboratory (Bar Harbor, ME, USA). Gender matched 8-12 week old mice were used for experiment. The mice received subcutaneous (s.c.) injection of B16-F10 cells (1.0 x 10^6^ cells in 100 μL of PBS) to the dorsal skin. The tumor volume was measured after 14 days of B160F10 injection. During the experimental period, the mice received intraperitoneal (i.p.) injection of creatine (500 mg/kg in 100 µl of saline), β-GPA (500 mg/kg in 100 μl f PBS), or saline (100 µl as a control). The tumor volume (V) was calculated by following a formula: V=(LxW^2^) x 0.52 (L>W).

### Macrophage stimulation assay

Macrophages (1.0 x 10^6^ cells/mL) were seeded in DMEM complete medium (D10, DMEM supplemented with 10% fetal bovine serum (FBS) and 1% penicillin-streptomycin) or B16-F10 cultured medium (CM) (D10 base) and were treated with control (PBS) or creatine (6.7 mM) at 37°C for 24 h. The CM and cells were subjected to ELISA and flow cytometry, respectively.

### Antigen uptake assay

Macrophages (1.0 x 10^6^ cells/mL) were seeded in D10 medium supplemented with control (PBS) or creatine (6.7 mM). FITC labeled-tyrosinase-related protein 2 (TRP-2) (100 μg/mL) was added to the cultures and the cells were incubated at 37°C for 24 h. TRP-2 uptake in macrophages was analyzed using flow cytometry.

### Ag presentation assay

Macrophages (1.0 x 10^6^ cells/mL) were mixed with tumor-sensitized (TS)-CD8+ T cells (5.0 x 10^6^ cells/mL) isolated from inguinal lymph nodes (LNs) of tumor-bearing mice (at day 7 of B16-F10 inoculation) in RPMI complete medium (R10, RPMI1640 supplemented with 10% FBS and 1% penicillin-streptomycin) containing TRP-2 peptide (10 μg/mL). The cultures were further treated with control (PBS) or creatine (6.7 mM) and were incubated at 37°C for 16 h, then the CD8+ T cells were analyzed using flow cytometry.

### Cytotoxity assay

B16-F10 cells (1.0 x 10^6^ cells/mL, target cells) were seeded with D10 medium and incubated at 37°C for overnight before assay. TS-CD8+ T cells (effector cells) were isolated from inguinal LNs of tumor-bearing WT mice (at day 7 of B16-F10 inculcation, from both saline- and creatine-treated mice) and applied to the B16-F10 culture at the indicated ration. The cultures were incubated at 37°C for 16 h, then the lactate dehydrogenase (LDH) level was measured in the CM using the Cytotoxicity LDH Assay Kit-WST (Dojin Chemical, Tokyo, Japan) by following the absorbance at 450 nm measured by microplate reader (TECAN M200; TECAN).

### Biochemical assay

Macrophages (1.0 x 10^6^ cells/mL) were cultured in D10 or B16-F10 CM supplemented with control (PBS) or creatine (6.7 mM) at 37°C for 24 h. The intracellular ATP and ADP levels were measured by using CellTiter-Glo 2.0 and ADP-Glo (Promega, Madison, WI, USA), respectively. Intracellular PCr level and CK activity were measured using the Mouse Creatine Phosphate (CP) ELISA Kit (MyBiosource, San Diego, CA, USA) and Creatine Kinase Activity Assay Kit (Sigma Aldrich), respectively. For CrT inhibition, the cultures were treated with β-GPA (10 mM). For inhibition of mitochondrial electron transport chain (ETC) and CK, the cultures were treated with oligomycin A (1 μg/mL) or BU99006 (50 μM) at 37°C for 6 h, respectively, then the cells were subjected to ATP assay. All procedures using assay reagents and kits were performed by following the product manuals.

### Glucose uptake assay

Macrophages (2.5 x 10^6^ cells/mL) were seeded in D10 medium or B16-F10 CM supplemented with control (PBS) or creatine (6.7 mM), and 2-(N-(7-Nitrobenz-2-oxa-1,3-diazol-4-yl) Amino)-2-Deoxyglucose (2-NBDG; 100 μM) was further added to the cultures. After incubation at 37°C for 24 h, the 2-NBDG uptake in macrophages was analyzed using flow cytometry.

### Statistics

Student’s t-test and one-way analysis of variance (ANOVA) were used to analyze the data for significant differences. Values of *p* < 0.05, *p* < 0.01, and *p* < 0.001 were regarded as significant.

## Results

### Creatine supplementation suppresses tumor growth accompanied by increased tumor-infiltrating inflammatory macrophages

**Figure 1:**
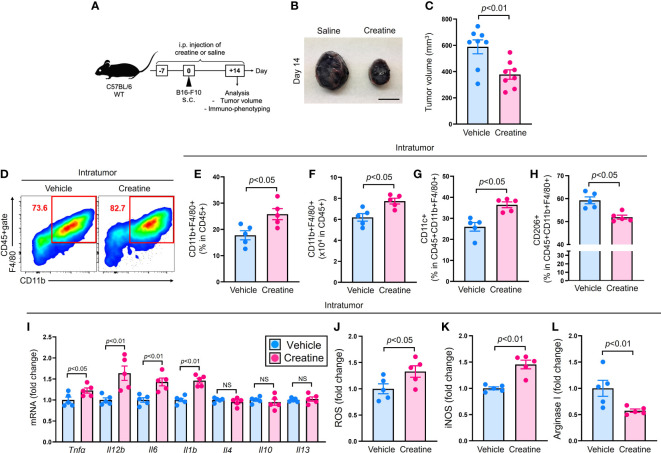
Creatine supplementation suppresses tumor growth accompanied with increased inflammatory IT macrophages. Experimental design of the murine tumor model. The WT (C57BL/6) mice received i.p. injection of saline (control) or creatine (500 mg/kg) daily from day -7 to +14 days. At day 0, B16-F10 cells (1.0x10^6^ cells/100 μl of PBS) were s.c. injected to the mice back skin. At day +14, the tumor volume was measured and IT immune cells were analyzed in each mouse using flow cytometry. **(B)** Representative pictures of formed tumors. **(C)** Cumulative values of tumor volumes. **(D)** Representative flow cytometry plots of IT macrophages. **(E, F)** Percentages **(E)** and numbers **(F)** of IT macrophages. **(G, H)** Percentages of CD11c+ **(G)** and CD206+ **(H)** IT macrophages. **(I)** Cytokines mRNA expressions in IT-isolated macrophages. **(J-L)** ROS **(J)**, iNOS **(K)**, and arginase I **(L)** production in IT macrophages. IT macrophages were defined as CD45+CD11b+F4/80+ in the flow cytometry analysis. The data are shown as mean +/- SEM of eight **(C)** or five (others) samples in two independent experiments. Student’s t-test was used to analyze data for significant differences. NS, non-significant.

**Figure 2:**
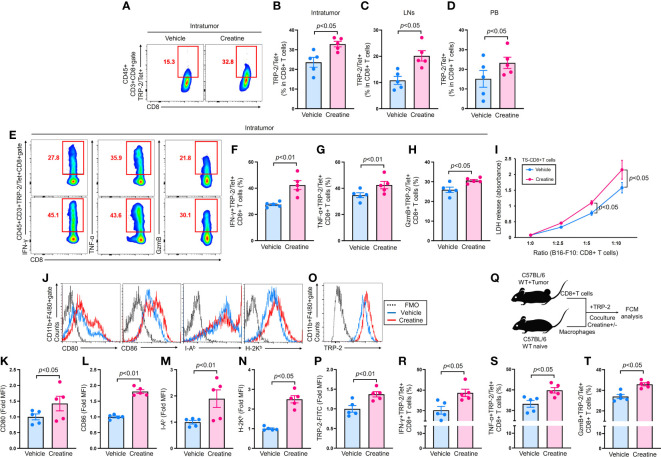
|Creatine supplementation increases anti-tumor CD8+ T cells by enhanced antigen presenting activity of macrophages. CD8+ T cells were analyzed in B16-F10-originated tumor bearing mice which were established by following the protocol described in **Figure 1A**. **(A)** Representative flow cytometry plots of IT TRP-2/Tet+CD8+ T cells. **(B–D)** Percentages of IT **(B)**, inguinal LNs **(C)**, and PB **(D)** TRP-2/Tet+CD8+ T cells. **(E)** Representative flow cytometry plots of IFN-γ, TNF-α, and granzyme B producing TRP-2/Tet+CD8+ T cells in the tumor. **(F–H)** Cumulative percentages of IFN-γ+ **(F)**, TNF-α+ **(G)**, and Granzyme B+ **(H)** TRP-2/Tet+CD8+ T cells in the tumor. **(I)** Cytotoxity assay using TS-CD8+ T cells against B16-F10 cells. TS-CD8+ T cells were isolated from inguinal LNs of tumor bearing mice (at day 7 of B16-F10 inoculation) and mixed with B16-F10 cells at the different ration indicated in the graph. After incubation at 37°C for 16 h, the cytotoxic activity of the TS-CD8+ T cells were assessed by LDH release (OD 450 nm) from B16-F10 cells. **(J–N)** Expression of antigen presentation related molecules on macrophages. Macrophages were cultured in B16-F10 CM with vehicle control (PBS) or creatine (6.7 mM) at 37°C for 24 h. The expression of CD80, CD86, I-A^b^, and H-2K^b^ were analyzed using flow cytometry. Representative histogram **(J)** and cumulative MFI values (fold change) of analyzed molecules **(K–N)**. **(O, P)** Antigen uptake of macrophages. TPMs were incubated with FITC-labeled TRP-2 (100 µg/mL) in D10 medium supplemented with vehicle control (PBS) or creatine (6.7 mM) at 37°C for 24 h. TRP-2 uptake was analyzed using flow cytometry. Representative histogram **(O)** and MFI values (fold change) **(P)** of incorporated FITC-labeled TRP-2 in macrophages. **(Q)** Experimental design of *in vitro* antigen presentation assay. TS-CD8+ T cells were isolated from inguinal LNs of tumor-bearing mice at day 7 of B16-F10 inoculation. The TS-CD8+ T cells were mixed with macrophages in the presence of TRP-2 (100 ng/mL). The cultures were further treated with vehicle control (PBS) or creatine (6.7 mM). After 16 h, the CD8+ T cells were analyzed using flow cytometry. The cumulative percentages of IFN-γ+ **(R)**, TNF-α+ **(S)**, or GzmB+ **(T)** TRP-2/Tet+CD8+ T cells were represented, respectively. TRP-2 (tumor Ag) specific CD8+ T cells were defined as CD45+CD3+TRP-2/Tet+CD8+ in the flow cytometry analysis. The cumulative data are shown as mean +/- SEM of five samples in two independent experiments. Student’s t-test was used to analyze data for significant differences.

To examine the effects of creatine supplementation on the immune response, we conducted an experiment using a mouse tumor model (**Figure 1A**). The mice were treated with either saline or creatine daily from day -7 to +14, and B16-F10 cells were injected into the skin. Tumor volumes were measured on day 14, and intratumor (IT) immune cells were analyzed using flow cytometry. Creatine supplementation significantly suppressed tumor growth compared with the control group. The average tumor volumes were 588.5 +/- 52.8 SEM mm^3^ for the control group and 377.6 +/- 35.8 SEM mm^3^ for the creatine-supplemented group (**Figures 1B, C**). Macrophages, the predominant myeloid cells in the tumor, increased in frequency and number in the tumors of the creatine-supplemented mice (**Figures 1D–F**). *In vitro* migration assays also demonstrated that macrophages exhibited enhanced chemotaxis towards B16-F10 cell-conditioned medium when treated with creatine (**Supplementary Figure 1**). Within the macrophage population, there was an increase in the CD11c+ M1 phenotype and a decrease in the CD206+ M2 phenotype with creatine supplementation (**Figures 1G, H**) (16). We assessed cytokine production by analyzing gene expressions in isolated macrophages from the tumor. The mRNA expressions of pro-inflammatory cytokines such as *Tnfa*, *Il12b*, *Il6*, and *Il1b* were significantly higher in the macrophages from creatine-supplemented mice compared with control mice. These increased pro-inflammatory cytokine levels were also observed at the protein levels in macrophages cultured with or without creatine (**Supplementary Figure 2**). However, the expression levels of typical anti-inflammatory cytokines, *Il4*, *Il10*, and *Il13*, which are predominantly produced by tumor-associated macrophages (TAM) in the tumor microenvironment (TME) (17), were similar between the control and creatine-supplemented groups (**Figure 2I**). Additionally, the production of macrophage-derived chemical mediators associated with the M1 phenotype, such as reactive oxygen species (ROS) and inducible nitric oxide synthase (iNOS), was significantly increased in macrophages from creatine-supplemented mice compared with control mice (**Figures 2J, K**) (17). Conversely, the immune-suppressive mediator arginase I, which is predominantly produced by the M2 phenotype, was downregulated with creatine treatment (**Figure 2L**) (18).

### Creatine supplementation promotes enhanced anti-tumor activity of CD8+ T cells via augmented antigen presenting activity of macrophages

CD8+ T cells are the main effector lymphocytes in anti-tumor immunity (19). To assess their activity in tumor-bearing mice, we detected tumor antigen-specific CD8+ T cells using an MHC class I tetramer loaded with TRP-2 peptide, a B16-F10 specific antigen (20). The tumors of creatine-supplemented mice exhibited a significant increase in TRP-2/Tetramer (Tet)+CD8+ T cells compared with controls (**Figures 2A, B**). Similarly, inguinal lymph nodes (LNs) and peripheral blood (PB) also showed an increase in TRP-2/Tet+CD8+ T cells due to creatine supplementation (**Figures 2C, D**). Moreover, creatine supplementation greatly enhanced the effector function of IT TRP-2/Tet+CD8+ T cells, as evidenced by increased IFN-γ, TNF-α, and granzyme B (GzmB) productions (**Figures 2E–H**). In a cytotoxicity assay, CD8+ T cells isolated from creatine-supplemented tumor-bearing mice exhibited higher killing ability against B16-F10 cells compared with CD8+ T cells from control mice (**Figure 2I**). We also investigated whether creatine supplementation enhanced antigen-presenting activity in macrophages. Macrophages cultured in B16-F10 CM showed significantly elevated levels of co-stimulatory molecules (CD80 and CD86) and antigen-presenting molecules (I-A^b^ and H-2K^b^) with creatine treatment compared to control (**Figures 2J–N**). Creatine treatment also promoted TRP-2 uptake by macrophages (**Figures 2O, P**). *In vitro* antigen presentation assays revealed that creatine-treated macrophages co-cultured with TS-CD8+ T cells resulted in larger percentages of IFN-γ+, TNF-α+, and GzmB+TRP-2/Tet+CD8+ T cells compared with controls (**Figures 2P–R**). Creatine did not alter CD8+ T cell activity, as IFN-γ production was comparable with and without creatine in CD8+ T cells stimulated by TCR-dependent stimulation (**Supplementary Figure 3**).

### Creatine supplementation increases ATP production in macrophages

**Figure 3:**
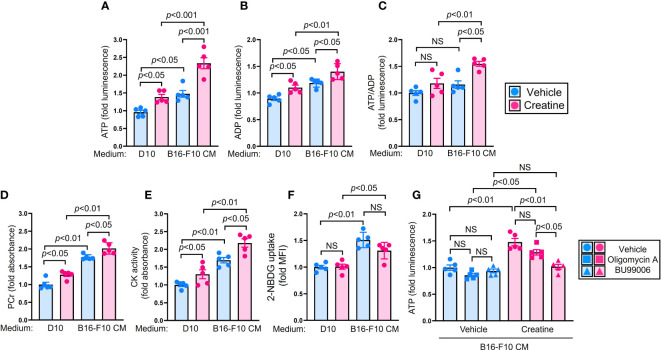
Creatine supplementation increases cellular ATP level in macrophages by utilizing phosphocreatine system. **(A–E)** Biochemical assay in macrophages. Macrophages were cultured in D10 or B16-F10 CM supplemented with vehicle control (PBS) or creatine (6.7 mM) at 37°C for 24 h. After incubation, the macrophages were used for each assay. Cellular ATP **(A)** and ADP **(B)** levels were measured in the macrophages by following the intensity of luminescence, respectively. **(C)** The ratio of cellular ATP/ADP was calculated from the assay results in **(A, B)**. **(D)** PCr level in the macrophages. **(E)** CK activity in macrophages. Both PCr and CK levels were calculated by following absorbance. **(F)** Glucose uptake assay in macrophages. Macrophages were cultured in D10, or B16-F10 CM supplemented with vehicle control (PBS) or creatine (6.7 mM). Fluorescence glucose analogue 2-NBDG (100 µM) was added to the cultures. After incubation at 37°C for 24 h, the 2-NBDG uptake in macrophages was analyzed using flow cytometry. The macrophage population was defined as CD11b+F4/80+ in flow cytometry analysis. **(G)** ATP assay with metabolic inhibition in macrophages. Macrophages were cultured in D10 medium, or B16-F10 CM supplemented with vehicle control (PBS) or creatine (6.7 mM). The cultures were further treated with vehicle control (PBS), oligomycin A (1 µg/mL), or BU99006 (50 µM), then incubated at 37°C for 6 h. The cellular ATP level was measured in macrophages by following the intensity of luminescence. The data are shown as mean +/- SEM of five samples in two independent experiments. One-way ANOVA was used to analyze data for significant differences. NS, non-significant.

We investigated the mechanism by which creatine enhances the functionality of macrophages. Firstly, we observed a significant increase in intracellular ATP levels upon creatine treatment in macrophages (**Figure 3A**). Furthermore, creatine treatment also led to an increase in intracellular ADP levels in macrophages (**Figure 3B**). The ATP/ADP ratio was found to be higher in macrophages treated with creatine, indicating an ATP-rich internal environment (**Figure 3C**). Creatine treatment resulted in elevated levels of PCr and enhanced CK activity in macrophages (**Figures 3D, E**). Interestingly, the effects of creatine treatment on measured substances and enzymatic activity were more pronounced in macrophages exposed to B16-F10 CM compared to those in D10 medium (**Figures 3A–E**). Creatine treatment did not increase glucose uptake in macrophages; instead, it was downregulated upon exposure to B16-F10 CM (**Figure 3F**). To identify the major metabolic pathway responsible for creatine-mediated ATP upregulation in macrophages, we used two different inhibitors. Oligomycin A specifically inhibits oxidative phosphorylation (OXPHOS) in mitochondria, while BU99006 inhibits the catalytic function of cytosolic CK (21, 22). The ATP upregulation induced by creatine treatment was not suppressed by oligomycin A. However, BU99006 completely abolished the ATP upregulation triggered by creatine treatment in macrophages (**Figure 3G**).

### Creatine transporter blockade abolishes creatine-mediated ATP upregulation and enhancing immune function of macrophages

**Figure 4:**
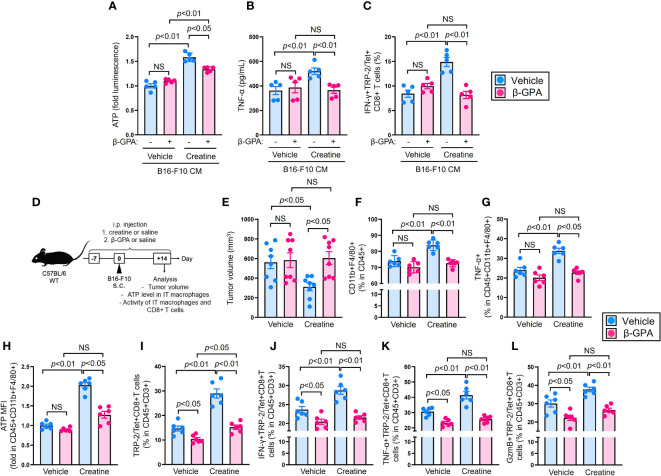
CrT blockade abolishes creatine supplementation-mediated enhancement of anti-tumor immunity in the mice. **(A, B)** Macrophage activity assay with CrT blockade. Macrophages were cultured in B16-F10 CM with or without β-GPA (10 mM), and further treated with vehicle control (PBS) or creatine (6.7 mM) at 37°C for 24 h. After incubation, cellular ATP level **(A)** and TNF-α production **(B)** were measured in the macrophages. **(C)** Ag presentation activity of macrophages with CrT blockade. Macrophages and TS-CD8+ T cells co-cultures were established by following the method represented in **Figure 2Q**. The cultures were treated with β-GPA and creatine by following the method shown in **Figure 4A**, **(B)** After incubation at 37°C for 16 h, the frequency of IFN-γ+TRP/Tet+CD8+ T cells were analyzed using flow cytometry. **(D)** Experimental design of CrT blockade in a murine tumor model. The WT (C57BL/6) mice received i.p. injection of saline (control) or β-GPA (500 mg/kg) as well as saline (control) or creatine (500 mg/kg) daily from day -7 to +14. At day 0, B16-F10 cells were inoculated by s.c. injection to the back skin of the mice, then tumor volume was measured and immune cells were analyzed in each mouse using flow cytometry at day +14. **(E)** Cumulative values of tumor volumes. **(F)** The percentages of IT macrophages. **(G)** TNF-α producing IT macrophages. **(H)** Intracellular ATP levels (fold MFIs) in IT macrophages. **(I–L)** Percentages of IT TRP-2/Tet+CD8+ T cells **(I)** and IFN-γ+ **(J)**, TNF-α+ **(K)**, and GzmB+ **(L)** in TRP-2/Tet+CD8+ T cells. The IT macrophages and TRP-2 (tumor Ag) specific CD8+ T cells were defined as CD45+CD11b+F4/80+ and CD45+CD3+TRP-2/Tet+CD8+, respectively, in the flow cytometry analysis. The cumulative data are shown as mean +/- SEM of eight **(A)** and six (others) samples in two independent experiments. One-way ANOVA was used to analyze data for significant differences. NS, non-significant.

To confirm whether extracellular creatine uptake directly increases ATP and enhances immunological activity in macrophages, we conducted CrT blocking experiments using β-guanidinopropionic acid (β-GPA), a competitive CrT blocker (23), in both *in vitro* and *in vivo* environments. Firstly, we examined the influence of CrT blockade on macrophages. The upregulation of ATP was suppressed in creatine-treated macrophages by β-GPA treatment (**Figure 4A**). TNF-α production did not increase with creatine treatment in the macrophages when CrT was blocked (**Figure 4B**). In the antigen presentation assay, β-GPA treatment failed to enhance Ag presentation activity in creatine-treated macrophages, resulting in comparable percentages of IFN-g+TRP-2/Tet+CD8+ T cells in the cultures, regardless of creatine presence (**Figure 4C**).

Next, we investigated the effect of CrT inhibition in the B16-F10 melanoma model. Mice were treated with β-GPA or saline (control) daily from day -7 to day +14. Additionally, the mice were treated with creatine or saline (control) following the same schedule shown in **Figure 1A**. B16-F10 cells were injected into the mice’s back skin on day 0, and tumor volumes were measured on day +14 (**Figure 4D**). Consistent with the results in **Figures 1B, C**, creatine supplementation significantly attenuated tumor growth compared to the control treatment in the mice (516.8+/-64.0 SEM mm^3^ vs 311.3+/-45.4 SEM mm^3^). However, CrT blockade failed to suppress tumor growth in the mice with creatine supplementation, and the tumor volumes were similar to the controls (582.8+/-87.3 SEM mm^3^ vs 604.4+/-75.9 SEM mm^3^) (**Figure 4E**). The percentage of tumor-infiltrating macrophages increased with creatine supplementation, while β-GPA-treated mice showed no difference in frequency between the control and creatine-supplemented groups (**Figure 4F**). Both TNF-α production and ATP levels were significantly increased in the intra-tumoral (IT) macrophages of the mice with creatine supplementation compared to those of the controls. However, creatine had no effect on ATP levels and TNF-α production in IT macrophages of β-GPA-treated mice (**Figures 4G, H**). Consistent with the modified macrophage activity, creatine supplementation increased the percentage of IT TRP-2/Tet+CD8+ T cells in the mice, but not in β-GPA-treated mice (**Figure 4I**). The production of IFN-γ, TNF-α, and granzyme B was also significantly increased in the IT TRP-2/Tet+CD8+ T cell population with creatine supplementation compared to the control in the mice. However, there were no differences in these effector CD8+ T cell populations between the β-GPA-treated mice with or without creatine supplementation (**Figures 4J–L**).

## Discussion

Macrophages primarily rely on mitochondrial oxidative phosphorylation (OXPHOS) for ATP production under normal circumstances (21, 22). However, in situations such as inflammation and the tumor microenvironment (TME), glycolysis and lipid degradation are also utilized to produce ATP, as immune responses require sufficient ATP levels (13, 21, 24). These metabolic pathways are quick and non-aerobic, allowing macrophages to rapidly generate ATP to meet their energy needs. However, these alternative pathways yield less ATP compared to OXPHOS and can limit the immune activation of macrophages due to insufficient ATP levels (21, 22). Promoting non-OXPHOS-mediated ATP production is a promising strategy to enhance the immunological activities of macrophages. Therefore, our focus is on creatine as a supplemental source for promoting ATP production. By investigating the effects of creatine on ATP synthesis and macrophage function, we aimed to determine its potential to enhance immune responses and improve the overall immunological activities of macrophages.

Our results clearly demonstrated that creatine supplementation enhances anti-tumor activity in mice, leading to a significant decrease in tumor volumes (**Figures 1B, C**). We observed two major differences in the immunological activities of creatine-supplemented tumor-bearing mice, namely, a promoted inflammatory phenotype of macrophages and enhanced activity of CD8+ T cells (**Figures 1G–L** and **Figures 2A–I**). These modified immune responses can be attributed to the increased ATP levels resulting from creatine supplementation in macrophages (**Figure 3A**). Importantly, we observed positive correlations between macrophage ATP levels and TNF-α production, as well as the frequency of IFN-γ+TRP-2+CD8+ T cells in the antigen-presentation assay (**Supplementary Figure 4**). Furthermore, we revealed that all functional upregulations in macrophages related to ATP increase were dependent on the uptake of extracellular creatine, as evidenced by CrT blockade (**Figure 4**).

Although our study has provided clear evidence that increased ATP levels enhance macrophage function through creatine supplementation, it is necessary to consider other unknown changes that may occur in macrophages as a result of creatine supplementation. While our focus was on the relationship between ATP production and immune response in macrophages, it is possible that creatine may induce other biological activities in these cells. Additionally, we need to consider the effects of creatine supplementation on other types of immune cells.

Interestingly, our findings differ from those of a previous report that also investigated the enhanced anti-tumor response resulting from creatine supplementation (15). In their study, the authors demonstrated that creatine supplementation attenuated tumor growth in mice inoculated with B16-F10-OVA cells. However, they concluded that the major cause of the anti-tumor immunity enhancement was increased ATP production in CD8+ T cells, rather than in macrophages or other antigen-presenting cells (APCs). In contrast, our study did not show any differences in CD8+ T cell activity with or without creatine supplementation during *in vitro* TCR-dependent stimulation without macrophages (**Supplementary Figure 3**). Furthermore, our analysis revealed the highest expression of *Slc6A8* (CrT) mRNA in macrophages, not in CD8+ T cells (**Supplementary Figure 5**), which supported our decision to focus on macrophages for creatine-based ATP upregulation. These discrepancies between our study and the previously published study may be attributed to various environmental factors, such as feeding sources and experimental settings. Additionally, the cell lines used for tumor formation differed between our experiment (B16-F10) and the previously published study (B16-OVA). To fully understand and reconcile these discrepancies and determine the true effects of creatine, further investigations from multiple perspectives and using different models are necessary.

To apply our findings to clinical settings, we must consider the various aspects of creatine. Creatine is a natural substance, and any excess is safely expelled into the external environment (25). Most previous studies have demonstrated that continuous creatine supplementation does not adversely affect kidney function (26, 27). However, there was one study that reported acute kidney failure resulting from continuous creatine supplementation at the recommended dosage (28). This evidence suggests that the dosage of creatine may need to be personalized within a suitable range for individuals to avoid critical side effects. Creatine has also been implicated as a negative factor in certain types of cancers. A study conducted on mouse models revealed that creatine promoted liver metastasis in colorectal and breast cancer (29). Another study demonstrated that inhibiting creatine metabolism impeded the growth and metastasis of prostate cancer (30). These findings pose a challenge to our melanoma results (15), indicating that the impact of creatine may be limited to specific cancer types in the functional modification of anti-tumor immunity. We hypothesized that creatine may effectively work for the tumor with abundant infiltrating macrophages. In murine melanoma models, we normally observed macrophages as a dominant population in the tumor infiltrating leukocytes. Further research is necessary to ascertain the safe use of creatine in enhancing anti-tumor immunity.

## Data availability statement

The raw data supporting the conclusions of this article will be made available by the authors, without undue reservation.

## Ethics statement

The animal study was reviewed and approved by Animal Welfare Committee of Jichi Medical University (Protocol No.; 20036-01, 20037-01) and University of South China (202005053).

## Author contributions

Conceptualization: ZP and SS. Methodology: ZP and SS. Experiments: ZP and SS. Formal analysis and investigation: ZP and SS. Discussion: ZP and SS. Writing – original draft: ZP and SS. Writing – review and editing: ZP and SS. Funding acquisition: ZP and SS. Resources: ZP and SS. Supervision: SS. All authors contributed to the article and approved the submitted version.
