# Supplementary material for: Creatine supplementation enhances anti-tumor immunity by promoting adenosine triphosphate production in macrophages
Source: Front Immunol. 2023 Aug 18;14:1176956. doi: 10.3389/fimmu.2023.1176956 (PMC10471797; doi:10.3389/fimmu.2023.1176956)
Supplement: Supplementary file 1 [file DataSheet_1.docx]

Supplementary Material

**Supplementary Figures**

**
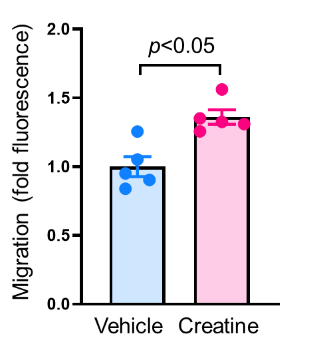
**

**Supplementary Figure 1. Creatine treatment promotes migration of macrophages**

CFSE stained macrophages (1.0 x 10^6^ cells/mL) were seeded in transmigration well with D10 medium supplemented with vehicle control (PBS) or creatine (6.7 mM), then migration activity toward to B16-F10 CM was assessed after incubation at 37℃ for 2 h. The data was shown as mean +/- SEM of five samples. Student *t*-test was used to analyze data for significant differences.

*
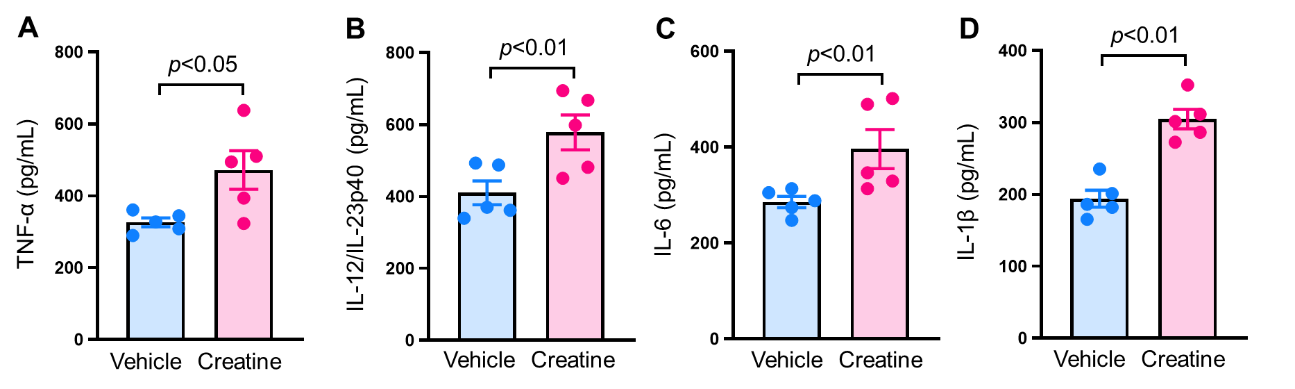
*

**Supplementary Figure 2. Creatine treatment increases pro-inflammatory cytokine production in macrophages**

Macrophages (1.0 x 10^6^ cells/mL) were seeded in B16-F10 CM, then were treated with vehicle control (PBS) or creatine (6.7 mM). After incubation at 37℃ for 24 h, cytokine concentrations in the cultured medium were measured by ELISA. The data were shown as mean +/- SEM of five samples. Student *t*-test was used to analyze data for significant differences.

**
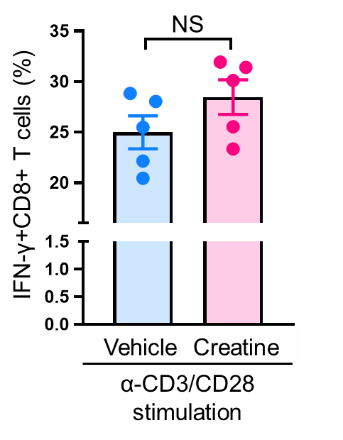
**

**Supplementary Figure 3. Creatine treatment does not alter CD8+ T cells activity**

Splenic CD8+ T cells (1.0x10^7^ cells/mL) were stimulated with anti-CD3/CD8 microbeads (1.0x10^7^ beads/mL) in R10 medium supplemented with vehicle control (PBS) of creatine (6.7 mM) at 37℃ for 72 h. The percentages of IFN-γ+CD8+ T cells were analyzed by flow cytometry. The data was shown as mean +/- SEM of five samples. Student *t*-test was used to analyze data for significant differences. NS: non-significant.


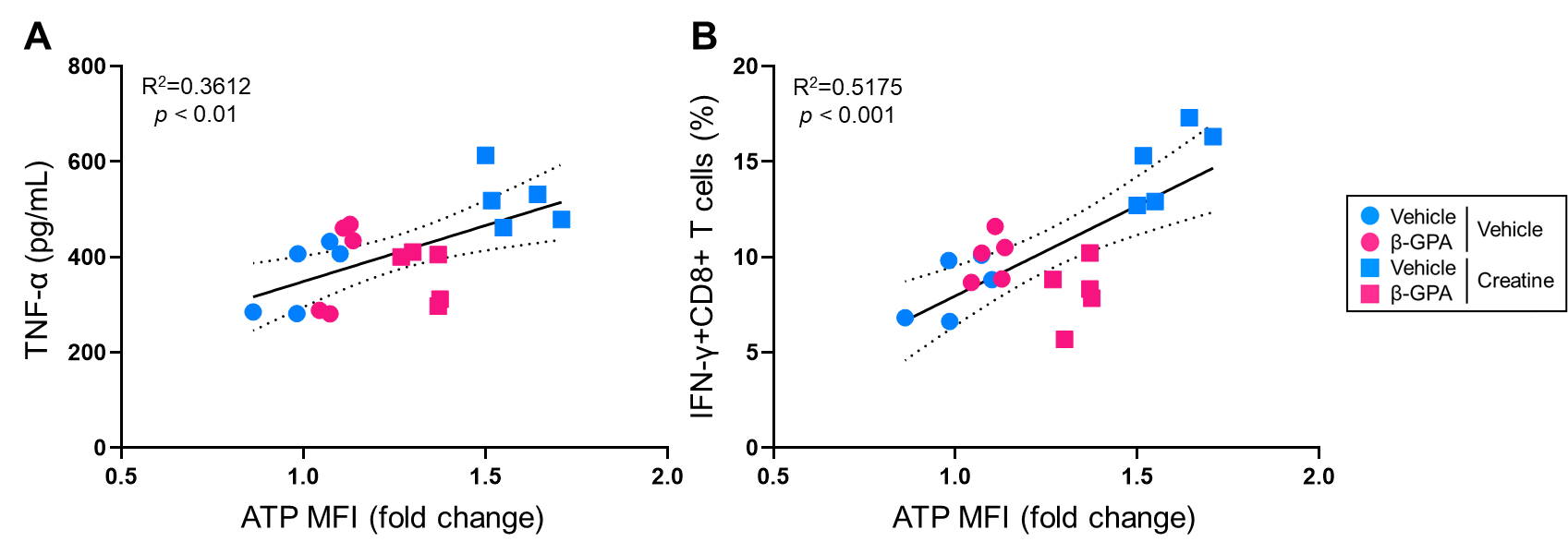


**Supplementary Figure 4. Correlation between ATP level and macrophages activity**

Correlations between two parameters were assessed by using the gained value of ATP levels (Figure 4A), TNF-α concentration (Figure 4B) and IFN-γ+TRP-2/Tet+CD8+ T cells (Figure 4C). The scattered plots and linear regressions were generated by GraphPad Prism 8.0 (GraphPad, San Diego, CA, USA). R^2^ and *p-*values were also calculated in the analyses.

**
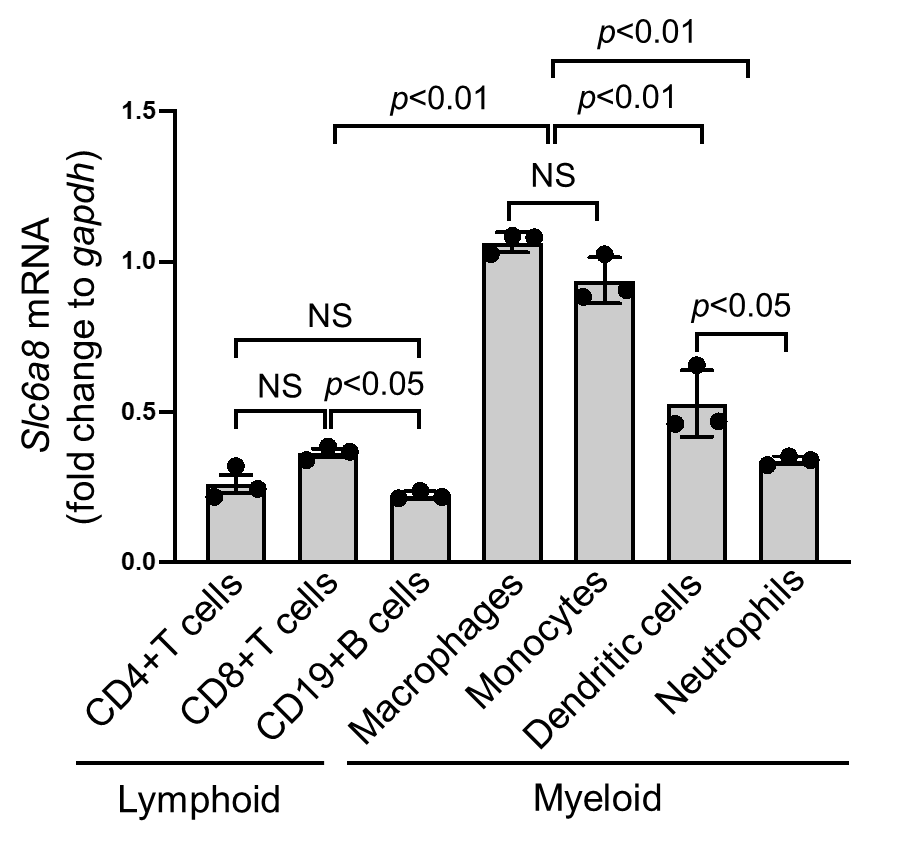
**

**Supplemental figure 5. Characterization of CrT mRNA expression in immune cells**

Total RNA was isolated from the indicated immune cells, and *Slec6a8* (CrT) mRNA expression levels were analyzed by real-time PCR. The *slec6a8* mRNA expression levels were normalized by *gapdh* mRNA expression in the corresponding samples. The data were shown as mean +/- SEM of three samples. One-way ANOVA was used to analyze data for significant differences. NS: non-significant.

**
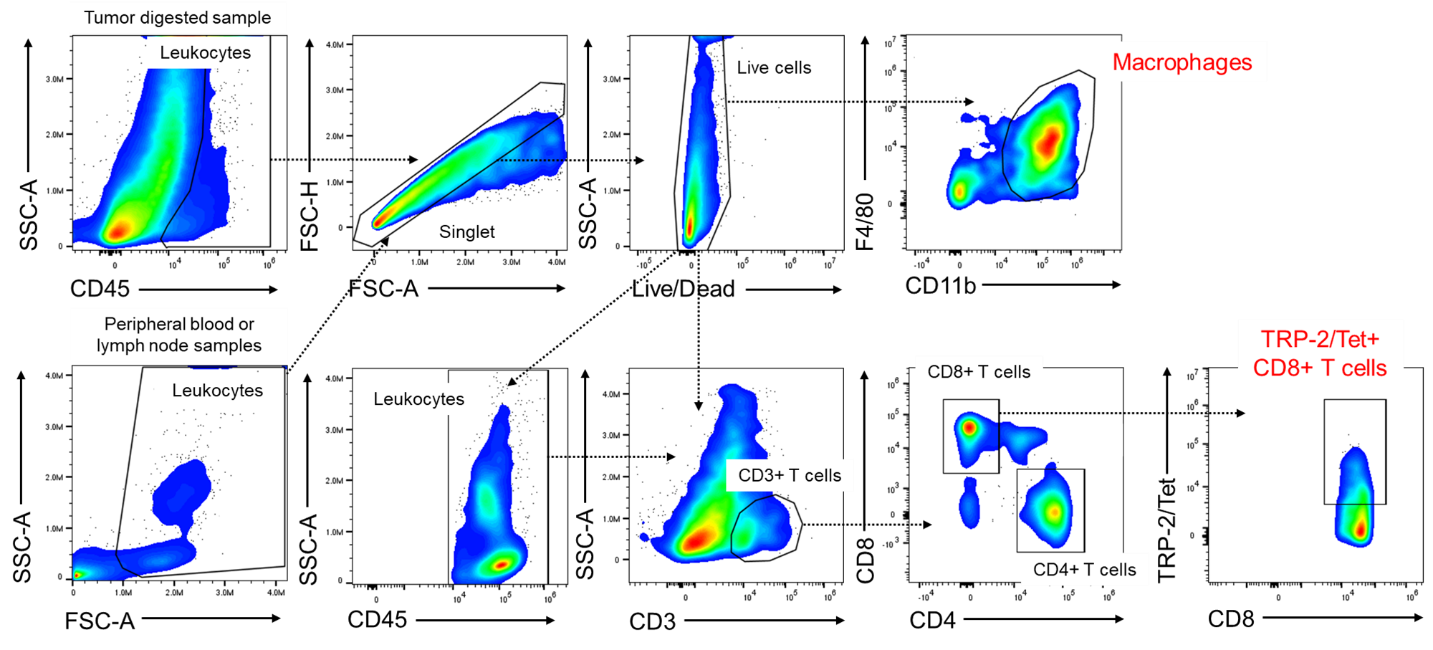
**

**Supplementary Figure 6. Gating strategy of flow cytometry**

**Supplementary Table 1. Primer sequences for real-time PCR**

*Tnfa*

Forward: 5’-GACGTGGAACTGGCAGAAGAG-3’

Reverse: 5’-TTGGTGGTTTGTGAGTGTGAG-3’

*Il12b*

Forward: 5’-TGGTTTGCCATCGTTTTGCTG-3’

Reverse: 5’-ACAGGTGAGGTTCACTGTTTCT-3’

*Il6*

Forward: 5’-TAGTCCTTCCTACCCCAATTTCC-3’

Reverse: 5’-TTGGTCCTTAGCCACTCCTTC-3’

*Il1b*

Forward: 5’-GCAACTGTTCCTGAACTCAACT-3’

Reverse: 5’-ATCTTTTGGGGTCCGTCAACT-3’

*Il4*

Forward: 5’-GGTCTCAACCCCCAGCTAGT-3’

Reverse: 5’-GCCGATGATCTCTCTCAAGTGAT-3’

*Il10*

Forward: 5’-GCTCTTACTGACTGGCATGAG-3’

Reverse: 5’-CGCAGCTCTAGGAGCATGTG-3’

*Il13*

Forward: 5’-CCTGGCTCTTGCTTGCCTT-3’

Reverse: 5’-GGTCTTGTGTGATGTTGCTCA-3’

*Slc6a8*

Forward: 5’-GTCTGGTGACGAGAAGAAGGG-3’

Reverse: 5’-CCACGCACGACATGATGAAGT-3’

*Gapdh*

Forward: 5’-AGGTCGGTGTGAACGGATTTG-3’

Reverse: 5’-TGTAGACCATGTAGTTGAGGTCA-3’

**Supplementary Methods**

**Breeding condition and ethical statement of animal experiment**

All of mice were bred and maintained in a specific pathogen-free (SPF) condition with 12 h day/night cycles and were allowed free access to food and water. Gender-matched adult mice (8-16 weeks) were used for each experiment. All animal experimental protocols were reviewed and approved by the Animal Welfare Committee of Jichi Medical University (Protocol No.; 20036-01, 20037-01) and University of South China (202005053).

**Reagents and peptide**

Creatine monohydrate, Phorbol 12-myristate 13-acetate (PMA), ionomycin, oligomycin A and 2-(Imidazolin-2-yl)-5-isothiocyanatobenzofuran (BU99006), β-Guanidinopropionic acid (β-GPA) and ATP-Red Live Cell Dye were purchased from Sigma Aldrich (St. Louis, MO, USA). BD Difco™ Thioglycolate Broth was purchased from BD bioscience (Franklin, NJ, USA). TRP-2 peptide (SVYDFFVWL) was synthesized by Peptide 2.0 (Chantilly, VA, USA). 2',7'-dichlorodihydrofluorescein diacetate (H2DCFDA), Fluorescein isothiocyanate (FITC) labeling kit, 5-(and 6)-Carboxyfluorescein diacetate succinimidyl ester (CFSE), 2-(N-(7-Nitrobenz-2-oxa-1,3-diazol-4-yl) Amino)-2-Deoxyglucose (2-NBDG) and anti-CD3/CD28 microbeads was purchased from Thermo Fisher Scientific (Waltham, MA, USA). Anti-CD3ε(145-2C11), andti-CD4 (GK1.5), anti-CD8 (53-6.7), anti-CD11b (M1/70), anti-CD45 (30-F11), anti-CD80 (16-10A1), anti-CD86 (GL-1), anti-F4/80 (BM8), anti-I-A^b^ (AF6-120.1), anti-H-2K^b^ (AF6-88.5), anti-IFN-γ (XMG1.2), anti-TNF-α (MP6-XT22), anti-Granzyme B (QA16A02), anti-NOS2 (iNOS) (W16030C), CD16/CD32 (2.4G2) were all purchased from Bio Legend (San Diego, CA, USA). Anti-Arginase I was purchased from R&D systems (Minneapolis, MN, USA). TRP-2/Tetrameter was purchased from MBL (Tokyo, Japen).

**Culture of B16-F10 cells**

B16-F10 cells were purchased from American Type Culture Collection (ATCC; Manassas, VA, USA). The frozen cell stock was thawed on ice, then the cells were washed with DMEM complete medium (D10; DMEM supplemented with 10% fetal bovine serum (FBS) and 1% penicillin-streptomycin). The cells were cultured in D10 medium at 37℃ until confluent. The B16-F10 cultured medium (CM) was collected and centrifuged at 300 g for 5 min, then was filtrated by 0.22 μm filter. The purified B16-F10 CM was stored at -80℃ until use.

**Tumor processing**

The excised tumor was copped by scissors in RPMI1640 medium (without FBS), then the sample was digested with collagenase (1 mg/mL) at 37 ℃ for 30 min. The sample was then filtrated on a 70 μm of cell strainer and residues were mechanically crushed on the filter. The isolated cells were washed with R10 medium and collected by centrifugation at 300 g for 5 min. The precipitated cells were used for analyses.

**Preparation of murine primary cells**

Extracted inguinal LNs and spleen were mechanically crushed on a 70 μm cell strainer with R10 medium, then the sample was centrifuged at 300 g for 5 min. After being washed the precipitated cells with R10 medium, the contaminated RBCs were eliminated by treatment with RBC lysis buffer (Thermo Fishier Scientific) at RT for 10 min. The samples were washed with R10 medium, then the cells were finally collected by centrifugation at 300 g for 5 min. The precipitated cells were used as LN cells and splenocytes. BM cells were flushed from tibia and femur in R10 medium using 10 ml syringe with 27G needle. The cells were washed with R10 medium followed by treating with RBC lysis buffer (Thermo Fisher Scientific) at RT for 10 min to eliminated RBCs. The sample was washed with R10 and the cells were collected by centrifugation at 300 g for 5 min. The precipitated cells were used as BM isolated cells. Peripheral blood was treated with RBC lysis buffer (Thermo Fisher Scientific) at RT for 10 min, then the sample was washed with PBS. After centrifuged at 300 g for 5 min, the precipitated cells were used as PB leukocytes. For antigen presentation assay, tumor-sensitized CD8+ T cells were isolated from the LN cells of tumor bearing mice (post day 7 of B16-F10 inoculation) by using mouse CD8+ T cell isolation kit. IT macrophages were isolated from melanoma processed cell suspension by using macrophage isolation kit. CD4+ T cells, CD8+ T cells, CD19 + B cells, CD11c+dendritic cells were isolated from splenocytes by using CD4+ T Cell Isolation Kit, CD8+ T cell Isolation kit, CD19 MicroBeads and CD11c MicroBeads UltraPure, respectively. Neutrophils and monocytes were prepared from BM isolated cells by using Neutrophil Isolation Kit and Monocyte Isolation Kit (BM), respectively. All isolation kits were purchased from Miltenyi Biotec (Bergisch Gladbach, North Rhine-Westphalia, Germany) and all procedures were performed by following the products manuals. The purities of isolated cells were analyzed by flow cytometry, and the samples with > 90 % of purified target population were used for subsequent experiments. All cell isolation procedures were performed by following the product manual of the kits.

**Peritoneal macrophage preparation**

For preparation of thioglycolate elucidated peritoneal macrophages (TPMs), the mice received an intraperitoneal (i.p.) injection of 3 ml of 3% thioglycolate (BD bioscience). After 84-96 h, the infiltrated leukocytes in the peritoneal cavity were collected and seeded on cell culture plate after being washed with R10 medium. The cells were incubated at 37℃ for 3 h, then the adherent cells were harvested as TPMs. The purity of TPMs was analyzed by flow cytometry. The samples with CD11b+F4/80+ > 90% were used for subsequent experiments.

**Migration assay**

Macrophages were stained with CFSE at 37℃ for 15 min. The CFSE-labeled macrophages (1.0 x 10^6^ cells/mL) were seeded in transmigration well (0.6 μm pore sized, Corning; Corning, NY, USA) with D10 medium supplemented with vehicle control (PBS) or creatine (6.7 mM). B16-F10 cultured medium (CM) was added in 96 well plate, and the transmigration well was placed on this plate. After incubation at 37℃ for 2 h, the transmigration well was detached and the migration activity was assessed by following fluorescence intensity originated from the CFSE-labeled migrated cells reading by microplate reader (TECAN M200; TECAN, Männedorf, Switzerland). The fluorescence signal from the migrated cells was calculated by following the formula; Fluorescence of migrated cells = Total fluorescence - (gravity effect + blank).

**Flow cytometry**

Flow cytometry analysis was performed by using LSRII (BD Biosciences). For extra cellular marker staining, the cells were first blocked with FcR blocker (anti-CD16/CD32) at 4℃ for 10 min followed by incubation with antibodies in PBS/2% FBS at 4℃ for 30 min. For intracellular staining, the sample was treated with BD Cytofix/CytoPerm Kit with GolgiStop^TM^ (BD Biosciences) by following a manufacture’s instruction. Briefly, the extracellular makers-stained cells were fixed and permeabilized with fixation buffer at 4℃ for 20 min. The fixed cells were stained with antibodies for intracellular target at 4℃ for 30 min. For intracellular cytokine detection in CD8+ T cells, the sample was re-stimulated with phorbol 12-myristate 13-acetate (PMA) (100 ng/mL) and ionomycin (250 ng/mL) in the presence of GolgiStop^TM^ at 37℃ for 6 h before staining. The antibodies and tetramer used in flow cytometry analysis were shown in reagents and antibodies. ROS production was analyzed by using H2DCFDA (Thermo Fisher Scientific). ATP level was analyzed by using ATP-Red Live Cell Dye (Sigma Aldrich). All analyses were performed by following the gating strategy represented in Supplementary Figure 6. All data were analyzed by BD FACS Diva (BD Bioscience) and FlowJo (BD Bioscience).

**Real-time PCR**

The total RNA was isolated from the cells by using TRIzol RNA Isolation Reagents (Thermo Fisher Scientific). The cDNA was synthesized from 250 ng of total RNA by using the PrimeScript RT reagent kit (TaKaRa, Tokyo, Japan). The expression level of mRNA was quantified by TB green system (TaKaRa) in Thermal Cycler Dice (TaKaRa). The primer sequences to specifically amplify target genes were indicated in Supplementary Table 1. The expression levels of target genes were normalized by following the expression of Gapdh mRNA expression. The comparative Ct method (ΔΔCt) was used for quantification of gene expression.

**Enzyme-linked immunosorbent assay (ELISA)**

Cytokine concentration was measured by Duo Set ELISA kit (R&D systems) for each target. All procedures were performed by following product manual. In B16-F10 CM treated sample, the actual produced cytokine concentration was calculated by following the formula; Actual cytokine concentration (C act) = total concentration in the culture (C total)- B16-F10 CM background (C back). All procedures were performed by following products manuals.
